# Supplementary material for: Conserving relics from ancient underground worlds: assessing the influence of cave and landscape features on obligate iron cave dwellers from the Eastern Amazon
Source: PeerJ. 2018 Mar 20;6:e4531. doi: 10.7717/peerj.4531 (PMC5865468; doi:10.7717/peerj.4531)
Supplement: Supplemental Information 1 [file peerj-06-4531-s001.pdf]

Supplemental Tables

Table S1: Summary of all cave and landscape metrics.

Variable	Description	Values (min / max)	Code
Species composition - MDS1	Scores calculated from Non-metric multidimensional scaling Axis 1 - continuous variable	-0.021 / 1.191	MDS1
Species composition - MDS2	Scores calculated from Non-metric multidimensional scaling Axis 2 - continuous variable	-0.013 / 1.424	MDS2
Species richness	The number of troglobites species	1.000 / 16.000	TrogRich
Rarity-weighted richness	Sum of the weighted number of species, where each species is divided by the total number of sites (caves) where it occurs	0.05 / 4.13	Wrich
Phylogenetic diversity	Minimum total length of all the phylogenetic branches required to span a given set of taxa on the phylogenetic tree from a set of species - continuous variable	789.999 / 6713.199	pd.obs
Highland	Canga highland representing geological units	-	Highland
Latitude	Decimal Geographic Coordinate System - continuous variable	-49.583, -50.383	Long
Latitude	Decimal Geographic Coordinate System - continuous variable	-5.893, -6.456	Lat
Altitude	The height above sea level where the cave is located (m). Vertical datum: Imbituba (SC) - continuous variable	224.000 / 842.000	Altitude
Horizontal length	The horizontal length of the cave from the entrance (m) - continuous variable	4.000 / 1546.000	HP
Slope	The steepness length of cave terrain (m) - continuous variable	0.100 / 38.200	Slope
Area	Cave area (m2) - continuous variable	7.000 / 4224.000	Area
Volume	Cave volume (m3) - continuos variable	5.000 / 4340.000	Volume
Percolating waters	Presence/absence of percolating waters - categorical variable	yes/no	PW
Water reservoirs	Presence/absence of water reservoirs - categorical variable	yes/no	WR
Vegetal material	Presence/absence of vegetal material - categorical variable	yes/no	Veg
Detritus	Presence/absence of detritus - categorical variable	yes/no	Det
Roots	Presence/absence of roots - categorical variable	yes/no	Root

Bat feces	Presence/absence of bat feces - categorical variable	yes/no	Guano
Other feces	Presence/absence of other feces - categorical variable	yes/no	Ofez
Regurgitation balls	Presence/absence of regurgitation balls - categorical variable	yes/no	RB
Carcasses	Presence/absence of carcasses - categorical variable	yes/no	Car
Bat populations	Presence/absence of bat populations - categorical variable	yes/no	BATPOP
Cave area vs buffer area - Base 50m	Sum of cave area within a radius of a buffer area of 50m (km^2) - continuous variable	0.001 / 1.000	area_vs_buff50
Cave area vs buffer area - Base 100m	Sum of cave area within a radius of a buffer area of 100m (km^2) - continuous variable	0.000 / 1.804	area_vs_buff100
Cave area vs buffer area - Base 250m	Sum of cave area within a radius of a buffer area of 250m (km^2) - continuous variable	0.000 / 1.586	area_vs_buff250
Cave area vs buffer area - Base 500m	Sum of cave area within a radius of a buffer area of 500m (km^2) - continuous variable	0.000 / 0.018	area_vs_buff500
Cave declivity 0- 10 (%)	The steepness length of cave terrain measured by laser scanning within a radius of a buffer area of 50m / 0-10 (%) - continuous variable	0.000 / 1.000	DCL_0_10B50
Cave declivity 1-20 (%)	The steepness length of cave terrain measured by laser scanning within a radius of a buffer area of 50m / 10-20 (%) - continuous variable	0.000 / 1.000	DCL_10_20B50
Cave declivity 20 - 30 (%) - Base 50m	The steepness length of cave terrain measured by laser scanning within a radius of a buffer area of 50m / 20-30 (%) - continuous variable	0.000 / 1.000	DCL_20_30B50
Cave declivity 0 - 10 (%) - Base 100m	The steepness length of cave terrain measured by laser scanning within a radius of a buffer area of 100m / 0-10 (%) - continuous variable	0.000 / 1.000	DCL_0_10B100
Cave declivity 10 - 20 (%) - Base 100m	The steepness length of cave terrain measured by laser scanning within a radius of a buffer area of 100m / 10-20 (%) - continuous variable	0.000 / 1.000	DCL_10_20B100
Cave declivity 20 - 30 (%) - Base 100m	The steepness length of cave terrain measured by laser scanning within a radius of a buffer area of 100m / 20-30 (%) - continuous variable	0.000 / 1.000	DCL_20_30B100
Cave declivity 0 - 10 (%) - Base 250m	The steepness length of cave terrain measured by laser scanning within a radius of a buffer area of 250m / 0-10 (%) - continuous variable	0.000 / 1.000	DCL_0_10B250

Cave declivity 10 - 20 (%) - Base 250m	The steepness length of cave terrain measured by laser scanning within a radius of a buffer area of 250m / 10-20 (%) - continuous variable	0.000 / 1.000	DCL_10_20B 250
Cave declivity 20 - 30 (%) - Base 250m	The steepness length of cave terrain measured by laser scanning within a radius of a buffer area of 250m /20-30 (%) - continuous variable	0.000 / 0.922	DCL_20_30B 250
Cave declivity 30 - 40 (%) - Base 250m	The steepness length of cave terrain measured by laser scanning within a radius of a buffer area of 250m / 30-40 (%) - continuous variable	0.000 / 0.004	DCL_30_40B 250
Cave declivity 0 - 10 (%) - Base 500m	The steepness length of cave terrain measured by laser scanning within a radius of a buffer area of 500m / 0-10 (%) - continuous variable	0.032 / 1.000	DCL_0_10B5 00
Cave declivity 10 - 20 (%) - Base 500m	The steepness length of cave terrain measured by laser scanning within a radius of a buffer area of 500m / 10-20 (%) - continuous variable	0.000 / 0.799	DCL_10_20B 500
Cave declivity 20 - 30 (%) - Base 500m	The steepness length of cave terrain measured by laser scanning within a radius of a buffer area of 500m / 20-30 (%) - continuous variable	0.000 / 0.620	DCL_20_30B 500
Cave declivity 30 - 40 (%) - Base 500m	The steepness length of cave terrain measured by laser scanning within a radius of a buffer area of 500m / 30-40 (%) - continuous variable	0.000 / 0.031	DCL_30_40B 500
Cave density - Base 50m	Sum of the number of caves within a radius of a buffer area of 50m - Km^2 - continuous variable	0.026 / 1.642	dens_50_km2
Cave density - Base 100m	Sum of caves the number of caves within a radius of a buffer area of 100m - Km^2 - continuous variable	0.012 / 0.615	dens_100_km 2
Cave density - Base 250m	Sum of the number of caves within a radius of a buffer area of 250m - Km^2 - continuous variable	0.003 / 0.248	dens_250_km 2
Cave density - Base 500m	Sum of the number of caves within a radius of a buffer area of 500m - Km^2 - continuous variable	0.001 / 0.109	dens_500_km 2
Cave distance to creeks (m)	The distance from the cave to the closest creek (m) - continuous variable	0.000 / 2526.422	dist_dreinage
Colluvial or flood - Base 50m	Geomorphology - Percentage of colluvial or flood terrain within a radius of a buffer area of 50m - continuous variable	0.000 / 100.000	CFLOOD_50
Steep slope of erosion - Base 50m	Geomorphology - Percentage of steep slope of erosion terrain within a radius of a buffer area of 50m - continuous variable	0.000 / 100.000	SSLOPE_50

Homogeneous or sharp differential - Base 50m	Geomorphology - Percentage of Homogeneous or sharp differential terrain within a radius of a buffer area of 50m - continuous variable	0.000 / 100.000	HSHARP_50
Homogeneous or convex differential - Base 50m	Geomorphology - Percentage of Homogeneous or convex differential terrain within a radius of a buffer area of 50m - continuous variable	0.000 / 100.000	HCONV_50
Homogeneous or tabular differential - Base 50m	Geomorphology - Percentage of Homogeneous or tabular differential terrain within a radius of a buffer area of 50m - continuous variable	0.000 / 100.000	HTAB_50
Buried pediplain degraded - Base 50m	Geomorphology - Percentage of Buried pediplain terrain degraded within a radius of a buffer area of 50m - continuous variable	0.000 / 1.000	PEDDEG_50
Buried pediplain retocaded - Base 50m	Geomorphology - Percentage of Buried pediplain retocaded terrain within a radius of a buffer area of 50m - continuous variable	0.000 / 100.000	PEDRET_50
Waterbody - Base 50m	Geomorphology - Percentage of Waterbody within a radius of a buffer area of 50m - continuous variable	0.000 / 99.636	WBODY_50
Colluvial or flood - Base 100m	Geomorphology - Percentage of colluvial or flood terrains terrain within a radius of a buffer area of 100m - continuous variable	0.000 / 100.000	CFLOOD_100
Steep slope of erosion - Base 100m	Geomorphology - Percentage of Steep slope of erosion terrain within a radius of a buffer area of 100m - continuous variable	0.000 / 100.000	SSLOPE_100
Homogeneous or sharp differential - Base 100m	Geomorphology - Percentage of Homogeneous or sharp differential terrain within a radius of a buffer area of 100m - continuous variable	0.000 / 100.000	HSHARP_100
Homogeneous or convex differential - Base 100m	Geomorphology - Percentage of Homogeneous or convex differential terrain within a radius of a buffer area of 100m - continuous variable	0.000 / 100.000	HCONV_100
Homogeneous or tabular differential - Base 100m	Geomorphology - Percentage of Homogeneous or tabular differential terrain within a radius of a buffer area of 100m - continuous variable	0.000 / 100.000	HTAB_100
Buried pediplain degraded - Base 100m	Geomorphology - Percentage of Buried pediplain degraded terrain within a radius of a buffer area of 100m - continuous variable	0.000 / 18.230	PEDDEG_100
Buried pediplain retocaded - Base 100m	Geomorphology - Percentage of Buried pediplain retocaded terrain within a radius of a buffer area of 100m - continuous variable	0.000 / 100.000	PEDRET_100
Waterbody - Base 100m	Geomorphology - Percentage of Waterbody within a radius of a buffer area of 100m - continuous variable	0.000 / 68.880	WBODY_100

Colluvial or flood - Base 250m	Geomorphology - Percentage of Colluvial or flood terrain within a radius of a buffer area of 250m - continuous variable	0.000 / 77.319	CFLOOD_250
Steep slope of erosion - Base 250m	Geomorphology - Percentage of Steep slope of erosion terrain within a radius of a buffer area of 250m - continuous variable	0.000 / 100.000	SSLOPE_250
Homogeneous or sharp differential - Base 250m	Geomorphology - Percentage of Homogeneous or sharp differential terrain within a radius of a buffer area of 250m - continuous variable	0.000 / 100.000	HSHARP_250
Homogeneous or convex differential - Base 250m	Geomorphology - Percentage of Homogeneous or convex differential terrain within a radius of a buffer area of 250m - continuous variable	0.000 / 100.000	HCONV_250
Homogeneous or tabular differential - Base 250m	Geomorphology - Percentage of Homogeneous or tabular differential terrain within a radius of a buffer area of 250m - continuous variable	0.000 / 100.000	HTAB_250
Buried pediplain degraded - Base 250m	Geomorphology - Percentage of Buried pediplain degraded terrain within a radius of a buffer area of 250m - continuous variable	0.000 / 31.465	PEDDEG_250
Buried pediplain retocaded - Base 250m	Geomorphology - Percentage of Buried pediplain retocaded terrain within a radius of a buffer area of 250m - continuous variable	0.000 / 100.000	PEDRET_250
Waterbody - Base 250m	Geomorphology - Percentage of Waterbody within a radius of a buffer area of 250m - continuous variable	0.000 / 53.045	WBODY_250
Colluvial or flood - Base 500m	Geomorphology - Percentage of Colluvial or flood terrain within a radius of a buffer area of 500m - continuous variable	0.000 / 50.472	CFLOOD_500
Steep slope of erosion - Base 500m	Geomorphology - Percentage of Steep slope of erosion terrain within a radius of a buffer area of 500m - continuous variable	0.000 / 100.000	SSLOPE_500
Homogeneous or sharp differential - Base 500m	Geomorphology - Percentage of Homogeneous or sharp differential terrain within a radius of a buffer area of 500m - continuous variable	0.000 / 100.000	HSHARP_500
Homogeneous or convex differential - Base 500m	Geomorphology - Percentage of Homogeneous or convex differential terrain within a radius of a buffer area of 500m - continuous variable	0.000 / 100.000	HCONV_500
Homogeneous or tabular differential - Base 500m	Geomorphology - Percentage of Homogeneous or tabular differential terrain within a radius of a buffer area of 500m - continuous variable	0.000 / 100.000	HTAB_500
Buried pediplain degraded - Base 500m	Geomorphology - Percentage of Buried pediplain degraded terrain within a radius of a buffer area of 500m - continuous variable	0.000 / 32.100	PEDDEG_500

Buried pediplain retocaded - Base 500m	Geomorphology - Percentage of Buried pediplain retocaded terrain within a radius of a buffer area of 500m - continuous variable	0.000 / 100.000	PEDRET_500
Waterbody - Base 500m	Geomorphology - Percentage of Waterbody within a radius of a buffer area of 500m - continuous variable	0.000 / 33.252	WBODY_500
Bare soil/Pasture/Agriculture - Base 50m	Landcover - Percentage of Bare soil/Pasture/Agriculture within a radius of a buffer area of 50m - continuous variable	0.000 / 100.000	BPA_50
Forest - Base 50m	Landcover - Percentage of Forest within a radius of a buffer area of 50m - continuous variable	0.000 / 100.000	FOREST_50
Mining - Base 50m	Landcover - Percentage of Mining within a radius of a buffer area of 50m - continuous variable	0.000 / 100.000	MINING_50
Montane savanna - Base 50m	Landcover - Percentage of Montane savanna within a radius of a buffer area of 50m - continuous variable	0.000 / 100.000	MONTSAV_50
Unclassified - Base 50m	Landcover - Percentage of unclassified landcover within a radius of a buffer area of 50m - continuous variable	0.000 / 16.315	UNCLASS_50
Bare soil/Pasture/Agriculture - Base 100m	Landcover - Percentage of Bare soil/Pasture/Agriculture within a radius of a buffer area of 100m - continuous variable	0.000 / 99.872	BPA_100
Forest - Base 100m	Landcover - Percentage of Forest within a radius of a buffer area of 100m - continuous variable	0.000 / 100.000	FOREST_100
Mining - Base 100m	Landcover - Percentage of Mining within a radius of a buffer area of 100m - continuous variable	0.000 / 98.004	MINING_100
Montane savanna - Base 100m	Landcover - Percentage of Montane savanna within a radius of a buffer area of 100m - continuous variable	0.000 / 100.000	MONTSAV_100
Unclassified - Base 100m	Landcover - Percentage of unclassified landcover within a radius of a buffer area of 100m - continuous variable	0.000 / 34.262	UNCLASS_100
Bare soil/Pasture/Agriculture - Base 250m	Landcover - Percentage of Bare soil/Pasture/Agriculture within a radius of a buffer area of 250m - continuous variable	0.000 / 93.578	BPA_250
Forest - Base 250m	Landcover - Percentage of Forest within a radius of a buffer area of 250m - continuous variable	0.000 / 100.000	FOREST_250
Mining - Base 250m	Landcover - Percentage of Mining within a radius of a buffer area of 250m - continuous variable	0.000 / 74.167	MINING_250
Montane savanna -	Landcover - Percentage of Montane savanna	0.000 / 100.000	MONTSAV_2

Base 250m	within a radius of a buffer area of 250m - continuous variable		50
Unclassified - Base 250m	Landcover - Percentage of unclassified landcover within a radius of a buffer area of 250m - continuous variable	0.000 / 18.273	UNCLASS_2 50
Bare soil/Pasture/Agriculture - Base 500m	Landcover - Percentage of Bare soil/Pasture/Agriculture within a radius of a buffer area of 500m	0.000 / 83.902	BPA_500
Forest - Base 500m	Landcover - Percentage of Forest within a radius of a buffer area of 500m - continuous variable	0.000 / 100.000	FOREST_500
Mining - Base 500m	Landcover - Percentage of Mining within a radius of a buffer area of 500m - continuous variable	0.000 / 70.272	MINING_500
Montane savanna - Base 500m	Landcover - Percentage of Montane savanna within a radius of a buffer area of 500m - continuous variable	0.000 / 100.000	MONTSAV_5 00
Unclassified - Base 500m	Landcover - Percentage of unclassified landcover within a radius of a buffer area of 500m - continuous variable	0.000 / 10.273	UNCLASS_5 00
BIF - Base 50m	Lithology - Percentage of Banded Iron Formation (BIF) within a radius of a buffer area of 50m - continuous variable	0.000 / 100.000	BIF_50
Laterites - Base 50m	Lithology - Percentage of Laterites within a radius of a buffer area of 50m - continuous variable	0.000 / 49.548	LATER_50
Conglomerate - Base 50m	Lithology - Percentage of Conglomerate within a radius of a buffer area of 50m - continuous variable	0.000 / 14.273	CONGL_50
Basic Rocks - Base 50m	Lithology - Percentage of Basic Rocks within a radius of a buffer area of 50m - continuous variable	0.000 / 100.000	BROCK_50
Basement - Base 50m	Lithology - Percentage of crystalline basement rocks within a radius of a buffer area of 50m - continuous variable	0.000 / 2.342	BASEMT_50
No information - Base 50m	Lithology - Percentage of lithology's without information within a radius of a buffer area of 50m - continuous variable	0.000 / 100.000	LNOINFO_50
BIF - Base 100m	Lithology - Percentage of Banded Iron Formation (BIF) within a radius of a buffer area of 100m - continuous variable	0.000 / 100.000	BIF_100
Laterites - Base 100m	Lithology - Percentage of Laterites within a radius of a buffer area of 100m - continuous variable	0.000 / 24.448	LATER_100
Conglomerate - Base 100m	Lithology - Percentage of Conglomerate within a radius of a buffer area of 100m - continuous variable	0.000 / 28.474	CONGL_100

Basic Rocks - Base 100m	Lithology - Percentage of Basic Rocks within a radius of a buffer area of 100m - continuous variable	0.000 / 100.000	BROCK_100
Basement - Base 100m	Lithology - Percentage of crystalline basement rocks within a radius of a buffer area of 100m - continuous variable	0.000 / 12.623	BASEMT_100
No information - Base 100m	Lithology - Percentage of lithology's without information within a radius of a buffer area of 100m - continuous variable	0.000 / 100.000	LNOINFO_100
BIF - Base 250m	Lithology - Percentage of Banded Iron Formation (BIF) within a radius of a buffer area of 250m - continuous variable	0.000 / 100.000	BIF_250
Laterites - Base 250m	Lithology - Percentage of Laterites within a radius of a buffer area of 250m - continuous variable	0.000 / 7.679	LATER_250
Conglomerate - Base 250m	Lithology - Percentage of Conglomerate within a radius of a buffer area of 250m - continuous variable	0.000 / 41.714	CONGL_250
Basic Rocks - Base 250m	Lithology - Percentage of Basic Rocks within a radius of a buffer area of 250m - continuous variable	0.000 / 100.000	BROCK_250
Basement - Base 250m	Lithology - Percentage of crystalline basement rocks within a radius of a buffer area of 250m - continuous variable	0.000 / 29.380	BASEMT_250
No information - Base 250m	Lithology - Percentage of lithology's without information within a radius of a buffer area of 250m - continuous variable	0.000 / 100.000	LNOINFO_250
BIF - Base 500m	Lithology - Percentage of Banded Iron Formation (BIF) within a radius of a buffer area of 500m - continuous variable	0.000 / 100.000	BIF_500
Laterites - Base 500m	Lithology - Percentage of Laterites within a radius of a buffer area of 500m - continuous variable	0.000 / 5.198	LATER_500
Conglomerate - Base 500m	Lithology - Percentage of Conglomerate within a radius of a buffer area of 500m - continuous variable	0.000 / 50.444	CONGL_500
Basic Rocks - Base 500m	Lithology - Percentage of Basic Rocks within a radius of a buffer area of 500m - continuous variable	0.000 / 100.000	BROCK_500
Basement - Base 500m	Lithology - Percentage of crystalline basement rocks within a radius of a buffer area of 500m - continuous variable	0.000 / 40.907	BASEMT_500
No information - Base 500m	Lithology - Percentage of lithology's without information within a radius of a buffer area of 500m - continuous variable	0.000 / 100.000	LNOINFO_500

Table S2: Mean age estimates (in million years) for internal nodes of the troglobite phylogeny (Fig. S2).

Node Name	Age Estimate [Myr]
Protostamia	790
Ecdysozoa	728
Arthropoda	698.5
Lophotrochozoa	698
[Arachnida + Dilopoda]	642
[Chilopoda + Dilopoda]	524
Arachnida	494
Diplopoda	460
[Opiliones + Pseudoscorpiones]	459
Polydesmida	443
Opiliones	439
Insecta	424
Glomeridesmida	366
[Araneae + Schizomida]	362
[Malacotaca + Insecta]	355
[Hemiptera + Tysanura]	344
Collembola	337
Malacostraca	334
[Coleoptera + Hymenoptera]	325
Hemiptera	302
Entomobryomorpha	284
Coleoptera	277
[Carabidae + Copelatinae]	237.2
[Tenebrionoidea + Staphylinidae]	234.8
[Scydmaenidae + Histeridae]	228
Hymenoptera	145

Table S3: Summary of the models describing total species richness, troglobitic species richness and presence of rare troglobites in relation to the mining area surrounding caves.

Response variable	Predictor variable	Estimate	SE	z	p
Total species richness[a]	Mining area (50m)	0.01	0.05	0.21	0.84
	Mining area (100m)	0.00	0.04	-0.02	0.98
	Mining area (250m)	-0.01	0.02	-0.31	0.75
	Mining area (500m)	-0.02	0.03	-0.66	0.51
Troglobitic species richness[a]	Mining area (50m)	-0.05	0.05	-1.00	0.32
	Mining area (100m)	-0.02	0.04	-0.51	0.61
	Mining area (250m)	0.04	0.04	1.16	0.25
	Mining area (500m)	0.03	0.04	0.72	0.47
Presence of rare troglobites[b]	Mining area (50m)	-0.27	0.25	-1.09	0.28
	Mining area (100m)	-0.21	0.16	-1.32	0.19
	Mining area (250m)	-0.18	0.1	-1.77	0.08
	Mining area (500m)	-0.09	0.09	-0.93	0.35

Response variables are shown followed by the predictor variable included in each model (models contained a single predictor), estimates, standard errors (SE), *z*-values and *p*-values. All models accounted for spatial autocorrelation and unaccounted variation between study sites by keeping the highland were the caves were located as a random effect. Two out of the 473 caves were excluded from these analyses because they contained missing data in at least one field. [a] Species richness was modeled using GLMM with Poisson distributed errors. [b] Presence of rare troglobites was modeled using GLMM with Bernoulli distributed responses (logistic regressions).
