# Supplementary material for: Conserving relics from ancient underground worlds: assessing the influence of cave and landscape features on obligate iron cave dwellers from the Eastern Amazon
Source: PeerJ. 2018 Mar 20;6:e4531. doi: 10.7717/peerj.4531 (PMC5865468; doi:10.7717/peerj.4531)
Supplement: Supplemental Information 2 [file peerj-06-4531-s002.pdf]

Supplemental Figures

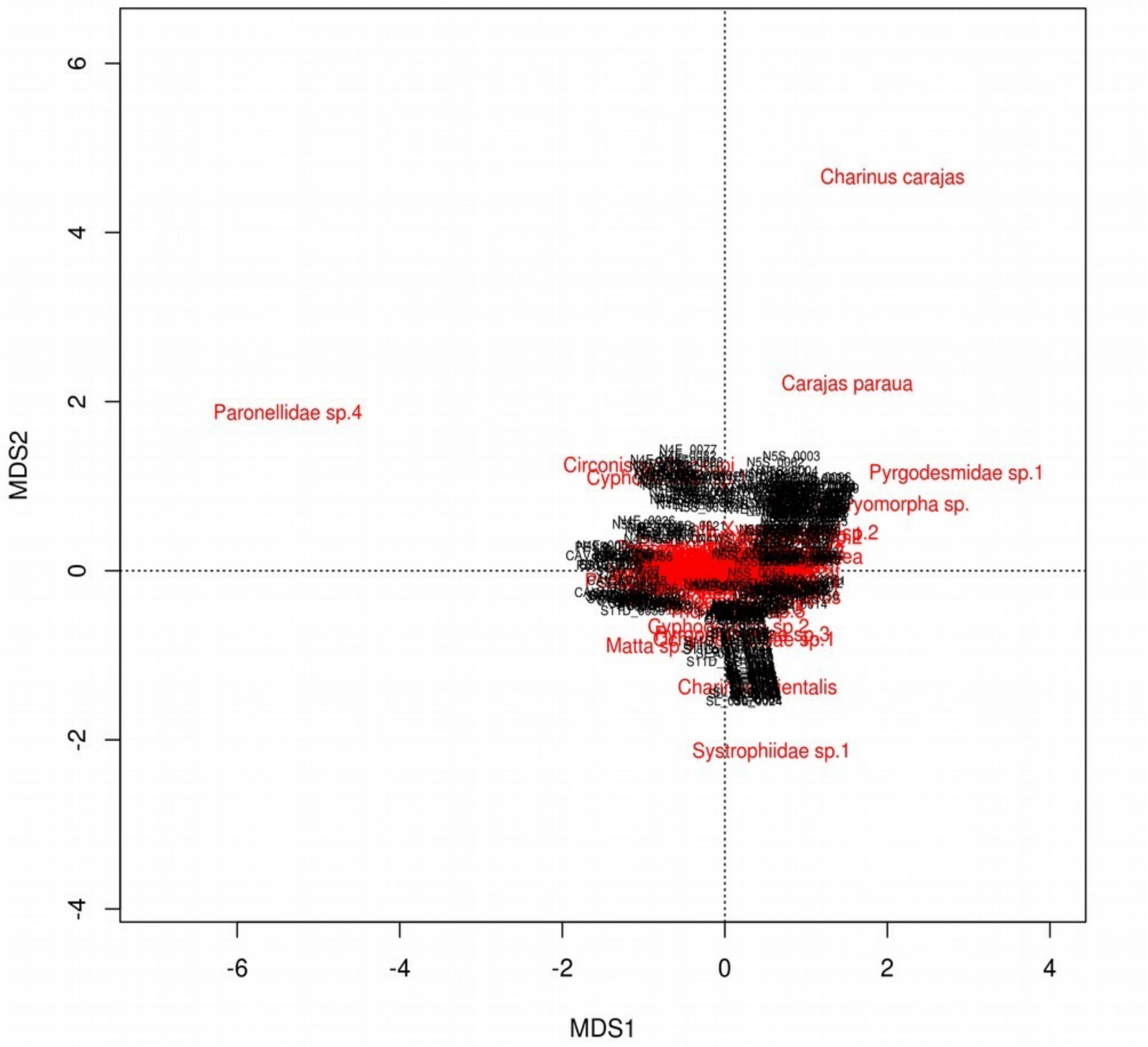

Figure S1: Principal coordinate analysis showing the distribution of some troglobitic species across the studied caves. Caves plotted in the same direction as specific troglobitic species actually contain those species.

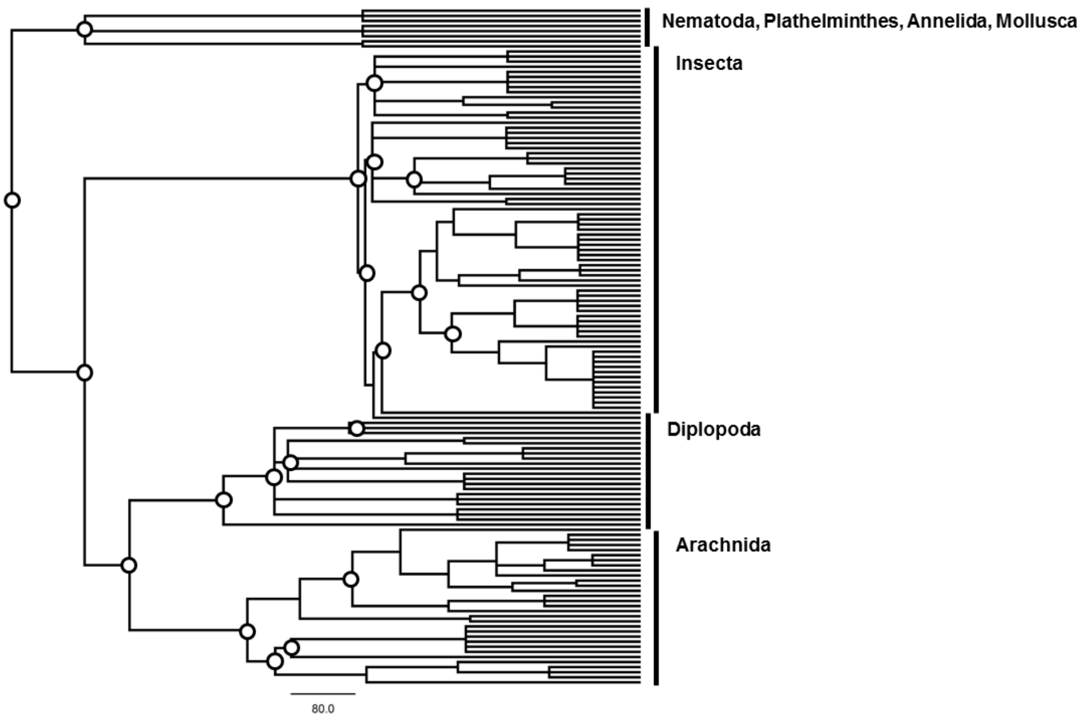

Figure S2: Phylogeny for the troglobitic species included in this study, retrieved from Hedges & Kumar (2009). Circles indicate dated nodes, while further nodes are evenly distributed among two dated nodes.

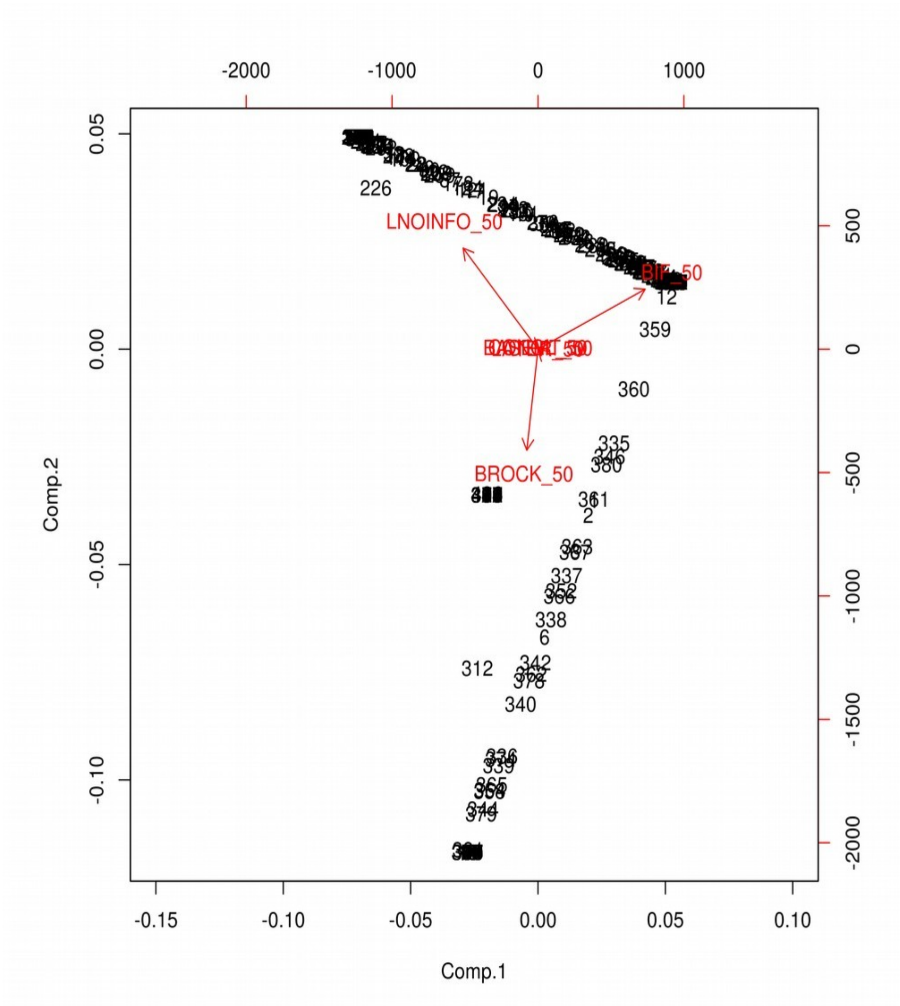

Figure S3: Plot showing the first two principal components of lithology variables (see Table S1 for full code legends).

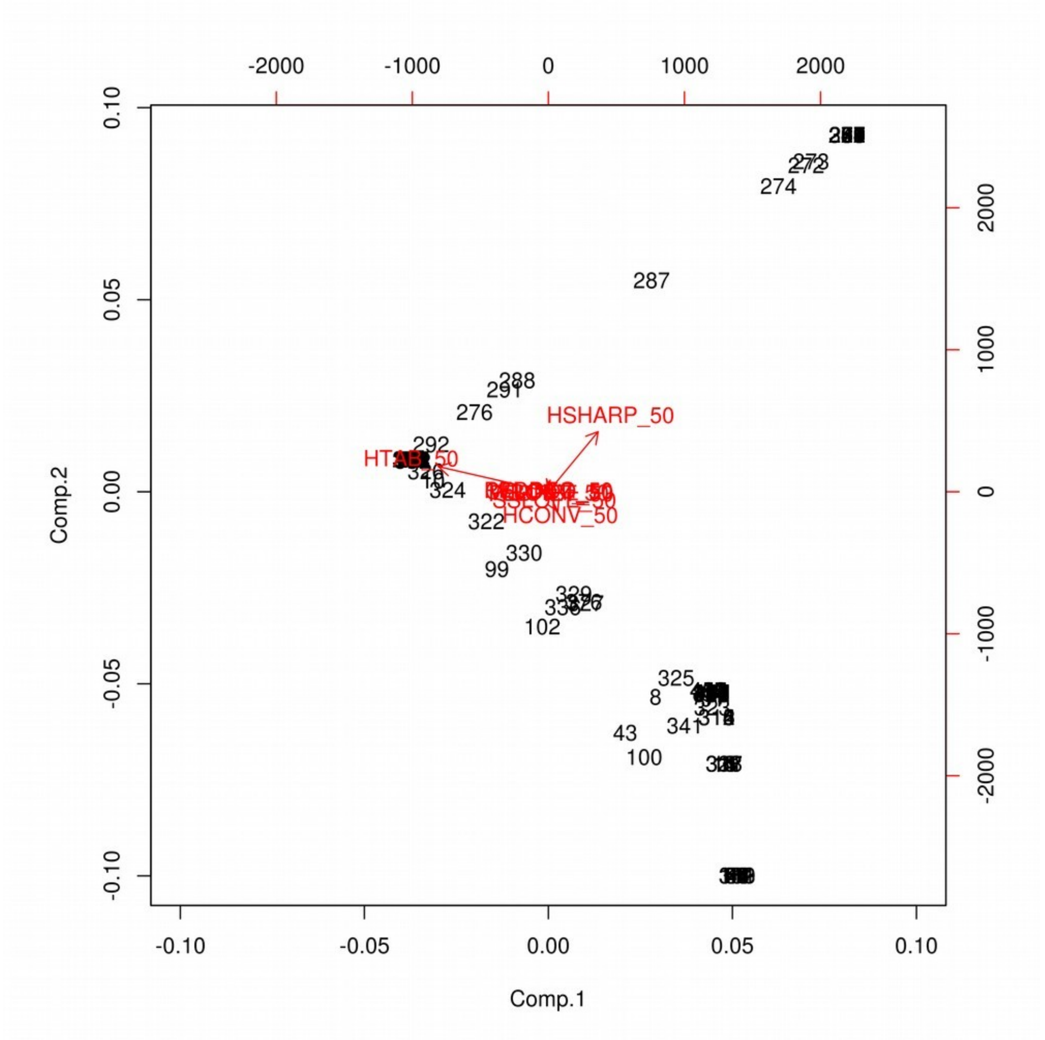

Figure S4: Plot showing the first two principal components of geomorphology variables (see Table S1 for full code legends).
